# Biocompatibility of Bespoke 3D-Printed Titanium Alloy Plates for Treating Acetabular Fractures

**DOI:** 10.1155/2018/2053486

**Published:** 2018-02-22

**Authors:** Xuezhi Lin, Xingling Xiao, Yimeng Wang, Cheng Gu, Canbin Wang, Jiahui Chen, Han Liu, Juan Luo, Tao Li, Di Wang, Shicai Fan

**Affiliations:** ^1^The Third Affiliated Hospital of Southern Medical University, Guangzhou 510600, China; ^2^School of Mechanical and Automotive Engineering, South China University of Technology, Guangzhou 510640, China

## Abstract

Treatment of acetabular fractures is challenging, not only because of its complicated anatomy but also because of the lack of fitting plates. Personalized titanium alloy plates can be fabricated by selective laser melting (SLM) but the biocompatibility of these three-dimensional printing (3D-printed) plates remains unknown. Plates were manufactured by SLM and their cytocompatibility was assessed by observing the metabolism of L929 fibroblasts incubated with culture medium extracts using a CCK-8 assay and their morphology by light microscopy. Allergenicity was tested using a guinea pig maximization test. In addition, acute systemic toxicity of the 3D-printed plates was determined by injecting extracts from the implants into the tail veins of mice. Finally, the histocompatibility of the plates was investigated by implanting them into the dorsal muscles of rabbits. The* in vitro* results suggested that cytocompatibility of the 3D-printed plates was similar to that of conventional plates. The* in vivo* data also demonstrated histocompatibility that was comparable between the two manufacturing techniques. In conclusion, both* in vivo* and* in vitro* experiments suggested favorable biocompatibility of 3D-printed titanium alloy plates, indicating that it is a promising option for treatment of acetabular fractures.

## 1. Introduction

Fracture of the acetabulum is a serve injury, due to its deep location and proximity to multiple blood vessels and nerves, and remains a challenge to trauma orthopedics [1]. According to the internal fixation doctrine of the Association for the Study of Internal Fixation (AO/ASIF), it is essential for a bone plate to provide strong internal fixation especially in acetabular fracture. Because of their outstanding mechanical properties and excellent biocompatibility, titanium alloy plates have been used to fix acetabular fractures for many years [2]. However, such titanium alloy plates are manufactured only in a fixed arcuate shape and require adjustment during surgery, which is time consuming and can put the patient at risk [3]. As a result, current plates cannot meet the needs of acetabular fracture patients and represent a barrier to further progress while the use of titanium in trauma cases has rapidly expanded in recent years. In common with other orthopedic applications [4], 3D-printing for acetabular fracture could provide a suitable solution.

**Figure 1:**
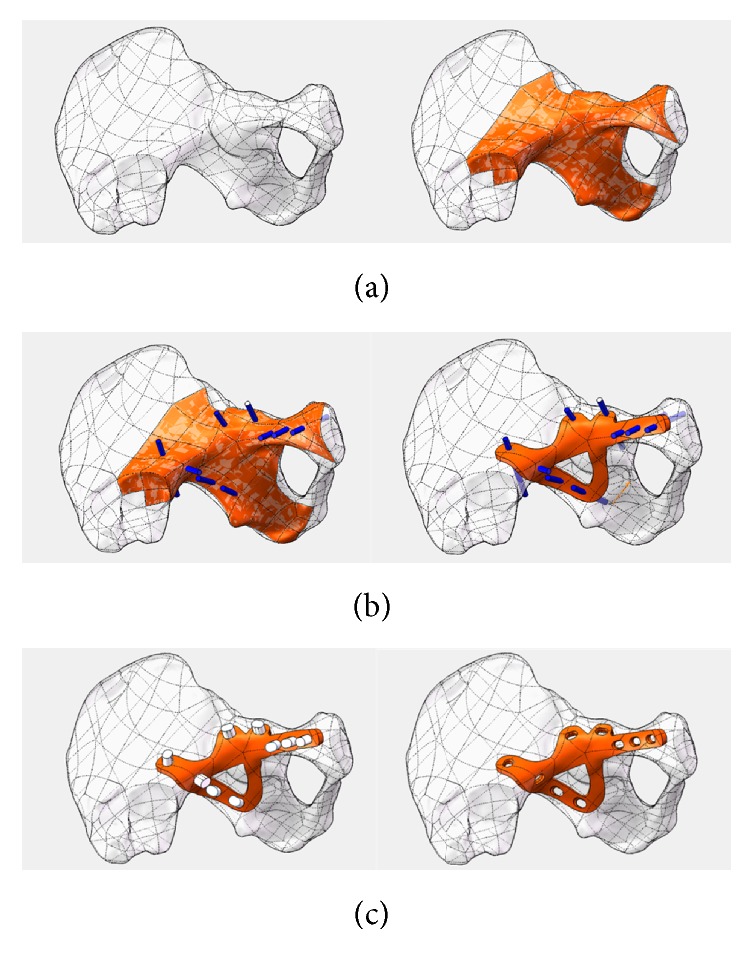
Plate individual designed by CAD software: (a) import CAD software to select the design surface; (b) precision set nails; thicken the surface to determine the shape of bone plate; (c) pressure hole direction to develop; get bone plate data.

Our previous study has reported a personalized plate for acetabular fractures, to improve the therapeutic effects of complex acetabular fractures [5] (Figure 1). The plate was manufactured by SLM, one of the most commonly used three- dimensional printing technologies. It was designed in the light of the specialties of the patient who suffered from high-energy injuries. The clinical data showed that the 3D-printed titanium alloy plates can effectively improve reduction quality and fixation effect.

**Figure 2:**
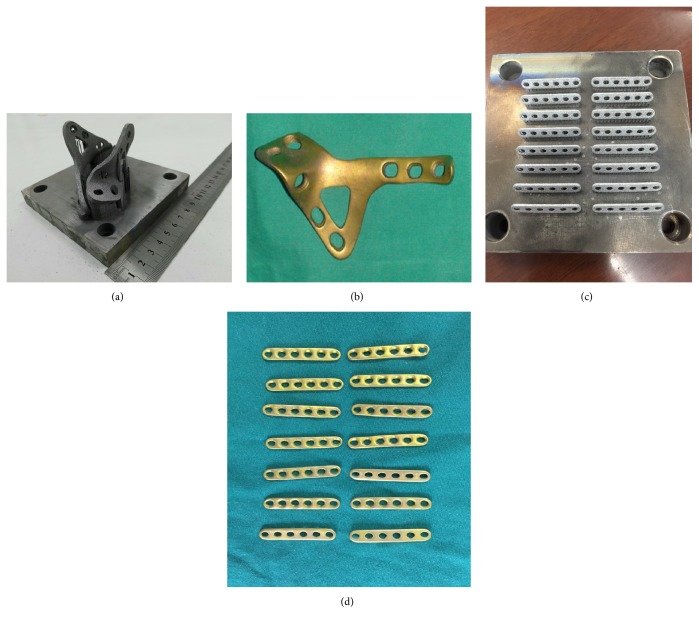
Plate manufactured via Selective Laser Melting: (a) workblank of 3D-printing titanium alloy (Ti6Al4V) plate; (b) personalized-design 3D-printing titanium alloy (Ti6Al4V) plate with series of standard postproduction modifications; (c, d) 3DP plates in the shape of conventional phalanx bone plate for* in vivo* and* vitro* study on the biocompatibility.

Titanium alloy (Ti6Al4V) has excellent properties including high specific strength, corrosion resistance, and biocompatibility, so is widely used in surgery [6]. In further research in using 3D-printing [7, 8], characterized by rapid manufacturing, of titanium alloy which has outstanding biocompatibility, individualized design plates could be a suitable alternative to treating complicated acetabular fracture. To date, many materials have been used for models of the acetabulum, but titanium alloys are only used in a limited number of 3D-printed implants of personalized design. In a preliminary study, we have already manufactured titanium alloy plates, personalized from a CT scan, via selective laser melting (SLM) and a series of standard postproduction modifications [9, 10] (Figure 2). Any novel manufacturing process requires proof of safety in humans before clinical application. Thus, the biocompatibility of 3D-printed plates requires testing using specific experiments [11, 12].

## 2. Experimental

### 2.1. Materials

3D-printed plates were manufactured using SLM by the SCTU, Guangdong Province, China, in the shape of a conventional phalanx bone plate for* in vivo* and* in vitro* biocompatibility studies (Figure 2). Dulbecco's modified Eagle's medium (DMEM) and fetal bovine serum (FBS) were purchased form Sigma-Aldrich (USA).

### 2.2. Animals

All animal experiments were approved by the Institutional Animal Care and Use Committee of the Third Affiliated Hospital of Southern Medical University.

Eighteen adult male New Zealand white rabbits (SMU Laboratory Animal Technology Co. Ltd, China) were housed in pairs in a bespoke clean room with food and water available ad libitum. Eighteen adult male albino guinea pigs and 30 adult male C57 mice of 18–20 g (SMU Laboratory Animal Technology Co. Ltd, China) were housed in aseptic padding and provided a standard pellet diet and water ad libitum.

### 2.3. Extracts of 3D-Printed Ti6AL4V Plates

3D-printed and conventionally manufactured plates were sterilized by high pressure according to ISO 10993-12:2007 [13], 121°C for 40 min, then incubated in FBS-containing DMEM at a ratio of 0.2 g/ml at 37°C in 5.0% CO_2_ for 24 h. Extracts were filtered to 0.22 *μ*m and those from 3D-printed specimens were diluted to 25%, 50%, and 100%.

### 2.4. Cytotoxicity Test

Extracts were divided into 5 groups: unused DMEM was a control (blank), extracts from conventional plates (NM group), and the three dilutions of 3D-printed extracts.

L929 mouse fibroblasts were purchased from Keygen Biotech Co. Ltd, China. They were cultured at a seeding density of 10^4^ cells/mL in 96-well plates in DMEM supplemented with 10% FBS and 1% antibiotics at 37°C in 5.0% CO_2_. Medium was changed every day until the cells were almost confluent. Culture medium was removed and 100 *μ*l of extract added to each well which was then incubated for 24, 72, and 120 hours. Four replicates were performed for each of the three time points. Ten *μ*L of CCK-8 solution (Dojindo Laboratories, Kumamoto, Japan) was added to each well then incubated at 37°C in 5.0% CO_2_ for 2 h in the dark. Cell morphology and population growth were evaluated using optical microscopy. Optical density of each well was measured at 450 nm using a plate reader (BioTek Multiscan Spectrum, USA). All data were expressed as mean ± standard deviation (SD). Relative growth rate (RGR) was calculated using the formula:

(1) $$\begin{array}{c} \text{RGR}=\frac{\left({\text{OD}}_{\text{3DPg}}-{\text{OD}}_{\text{blank}}\right)}{\left({\text{OD}}_{\text{NMg}}-{\text{OD}}_{\text{blank}}\right)}\times 100\%. \end{array}$$

See [13].

Statistical analysis was performed using 20.0 SPSS (USA). Differences between groups were analyzed by two-way ANOVA comparison test.

### 2.5. Dermal Irritation Test

Allergenicity was assed using a guinea pig maximization test. Six adult, male albino guinea pigs were allocated randomly into one of three groups: blank control, positive control, and 3D-printed group. Three required solutions were prepared before the test. Solution A comprised a 1 : 1 dilution of complete Freund's adjuvant (CFA, Sigma-Aldrich, USA), with physiological saline. On the basis of above groupings, three variations of Solution B were prepared, for each guinea pig group: normal saline (B1), 5% formaldehyde (B2), and 3D-printed plate extracts (B3). For each group Solution C was prepared from a 1 : 1 mixture of Solutions A and B. The day before the test, a 40*∗*50 mm section on the back of each guinea pig was shaved. The following day this area was disinfected using 75% alcohol, twice, and 3 points, a, b, and c, separated from each other by 15 mm were selected for intracutaneous injection. 0.1 ml each of Solution A and the correct Solutions B and C for the different guinea pig groups were injected into points a–c, for generating sensitization. The backs were shaved again on the seventh day and 10% SDS was injected into those points to strengthen the immune reaction. On the next day, gauze soaked in Solution B was laid on each back for 24 h, referred to as the topical induction phase. Fourteen days after the sensitization process, the abdomens of the animals were shaved and gauze soaked in Solution C fixed in place, for 24 h. This was referred to as the challenge phase. The condition of the dorsal and abdominal skin and animal movements were noted on consecutive days. After examination, each animal was executed and the abdominal and dorsal skin removed for pathological examination after fixation in 4% paraformaldehyde for 24 h. Sections were paraffin-embedded and hematoxylin and eosin (H & E) stained. Samples were evaluated for inflammation by microscopy and graded appropriately.

### 2.6. Acute Systemic Toxicity Test

Thirty male C57 mice, weighing 18–20 g, were randomly selected into three groups: negative control, conventional plate, and 3D-printed group. Weights of all animals were recorded. Extracts were created as described in Section 2.3, except that DMEM was replaced by normal saline and 0.1 ml injected intravenously into the tail. Mice were weighed for three consecutive days, and their movements and what they consumed and excreted were recorded after the injection. These data were analyzed statistically to evaluate the effect of plate extract on the mice. All mice were executed via spinal cord transection on the third day following the test. Kidney and liver samples were retained for pathological examination after H & E staining.

### 2.7. Muscle Implantation Test

Eighteen New Zealand white rabbits were randomly divided into two groups for the muscle implantation test. The animals were housed in pairs for several days prior to the experiment to allow them to adapt to their surroundings. The animals were anesthetized by Pentobarbital Sodium via the auricular vein at a dosage of 1 ml/kg and their backs shaved. The shaved area was disinfected twice with iodine solution. A longitudinal incision was made in the disinfected area and blunt dissection was performed to the dorsal lumbar muscles on each side of the spine. Conventional and 3D-printed plates were implanted into the muscles, contralaterally. The incision was closed using stitches and cleaned using normal saline. After surgery, the animals had intramuscular injections of 1.0 ml benzylpenicillin potassium (four hundred thousand units) every day for three days. The behavior of the animals, including activity and excreta, was recorded to evaluate the influence of the plates on them. The animals were sacrificed by injection of air via the auricular vein. Muscle tissue around the plate and the kidney and liver were explanted for histopathologic examination. Three different time periods: 1 w, 4 w, and 12 w, were examined, in accordance with the time corresponding to early, medium, and late phases after implantation. Tissues were fixed with 4% paraformaldehyde for approximately 24 h and then placed into an automatic biological-tissue hydroextractor for dehydration prior to paraffin-embedding. Tissue blocks were cut into 3 *μ*m slices for H & E staining followed by histopathologic examination.

## 3. Results

### 3.1. Cytotoxicity Test

**Figure 3:**
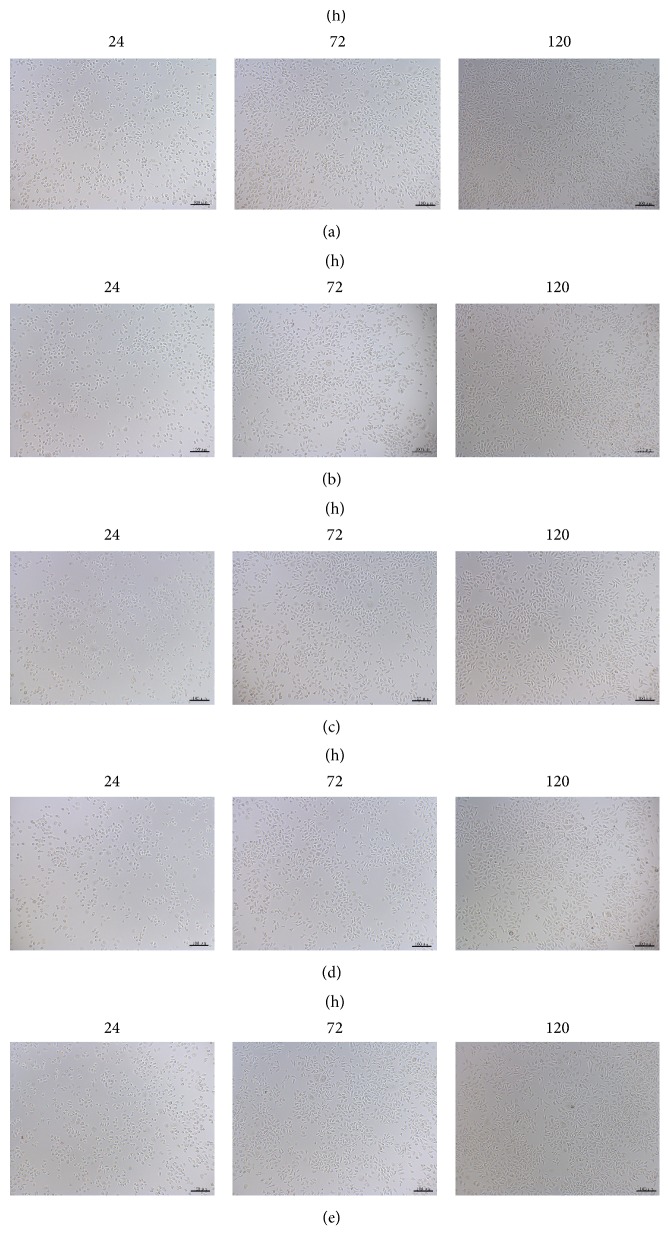
Cellular morphology via optical microscope at different times (100x): (a) group of 0% 3D-printing plate (blank group); (b) group of 25% 3D-printing plate; (c) group of 50% 3D-printing plate; (d) group of 100% 3D-printing plate; (e) group of NM plate.

**Figure 4:**
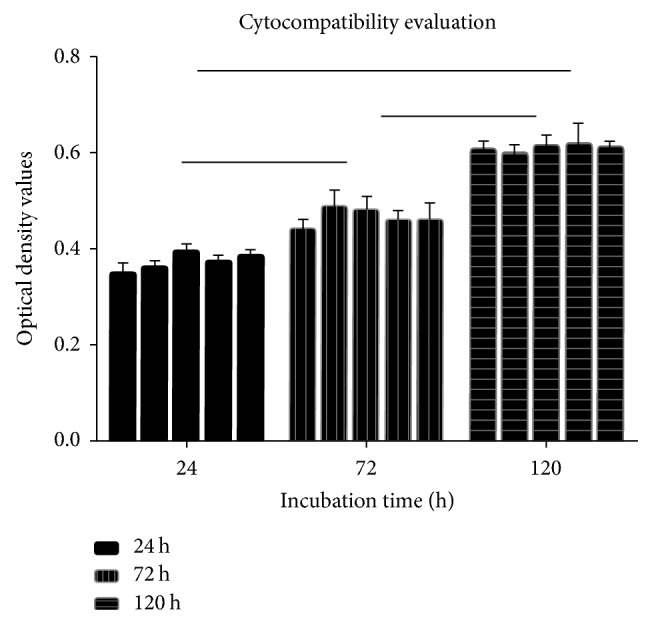
Cell OD values in each group at different times (hours).

As shown in Figure 3, no significant apoptosis was presented in all groups for the cell culture. Simultaneously, the CCK-8 assay revealed that the OD values of each group were increased gradually associated with the time prolonged (*P* < 0.05) (Figure 4 and Table 1). The RGR were shown in Table 2, ranging from 72.65% to 106.58% of 3D-printed extracts. These results suggested that the cytocompatibility of the 3D-printed plate is no less than that of the conventional plate.

### 3.2. Dermal Irritation Test

**Figure 5:**
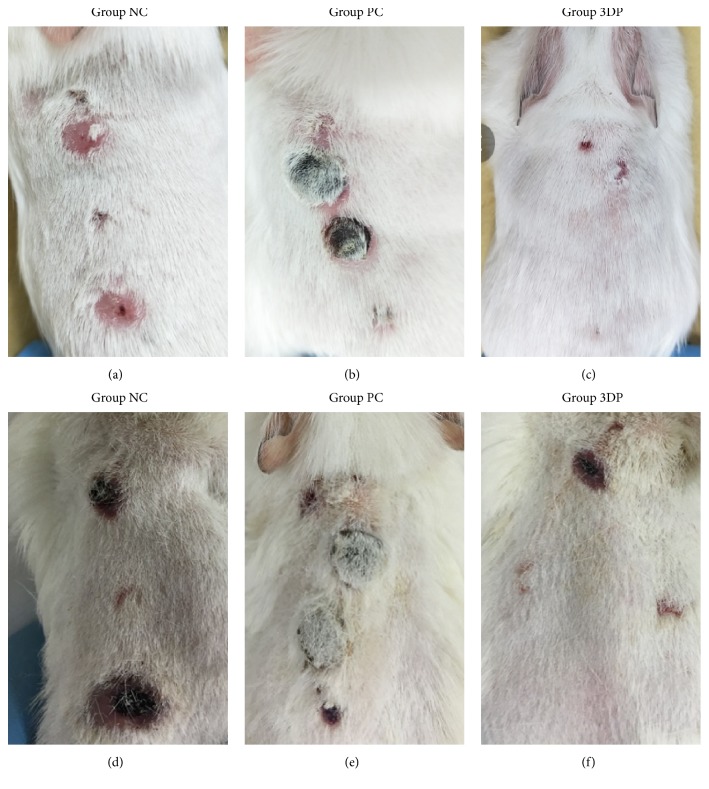
Observation on the skin conditions of the animals' backs, NC: negative control, PC: positive control: (a)~(c) sensitization process at ninth day; (d)~(f) excitation phase at nineteenth day.

**Figure 6:**
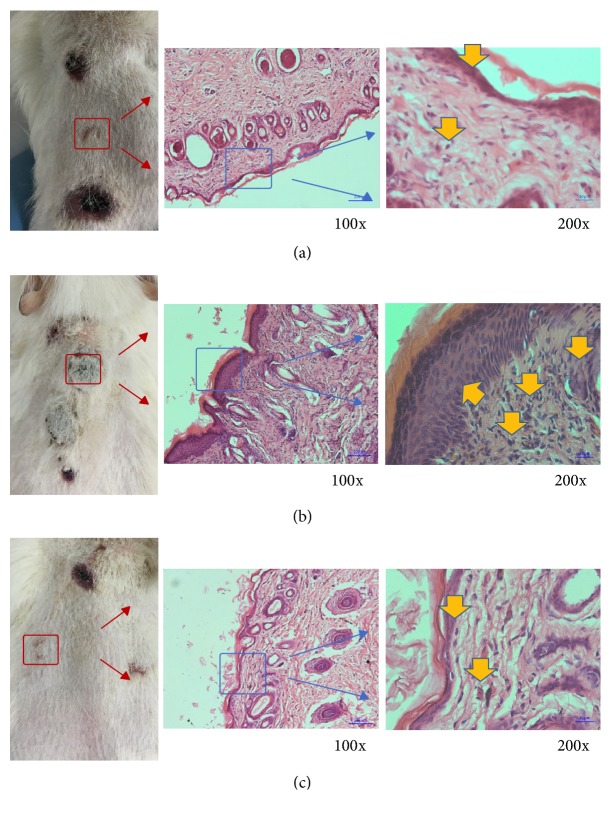
Pathological evaluation of three groups: (a) negative control group; (b) positive control; (c) 3D-printed plate group. Yellow arrows indicate some inflammatory cells.

During dorsal skin observation, point A reactions were similar among the three groups of guinea pigs with mild swelling after local induction. Point B reactions of the 3D-printed group were consistent with those in the blank control group, topical induction showing no obvious inflammatory reaction. Point B skin reaction showed a serious sensitization response observed in the positive control group. There was no obvious localized erythema in the abdominal skin of the 3D-printed or control groups, but the positive control group appeared necrotic and scabby (Figure 5). The results of pathological evaluation by light microscopy in the 3D-printed group were consistent with that of the blank control group, with no soakage of diffuse dermis or epidermal mononuclear cells and less inflammatory cell aggregation. Findings in the positive control group were all opposite to these, with cell necrosis surrounded by lymphocytes (Figure 6).

### 3.3. Acute Systemic Toxicity Test

**Figure 7:**
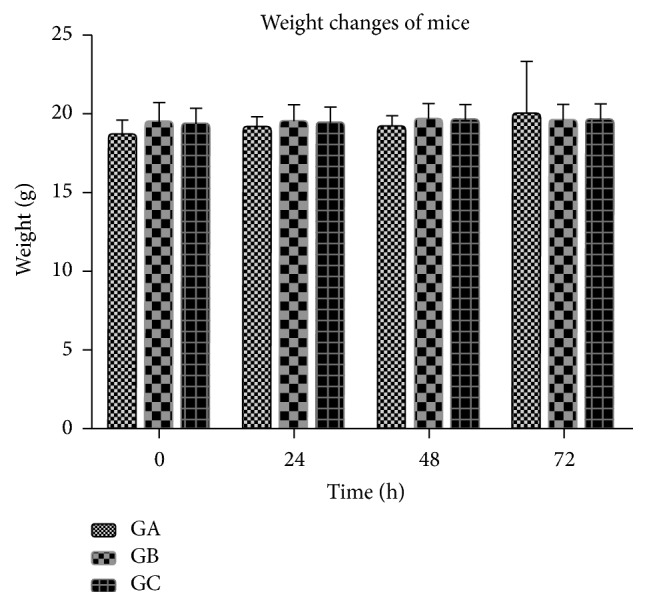
Changes of weight from three groups at four times, GA: 3DP group, GB: NM group, GC: negative control group.

Following intravenous injection into the tail, the activity, diet, and excretion of the mice were normal. The weight change of each group of mice was measured in 4 consecutive days after transplantation. Results showed that no significant difference of weight change was exhibited in each group at each point (Figure 7).

### 3.4. Muscle Implantation Test

**Figure 8:**
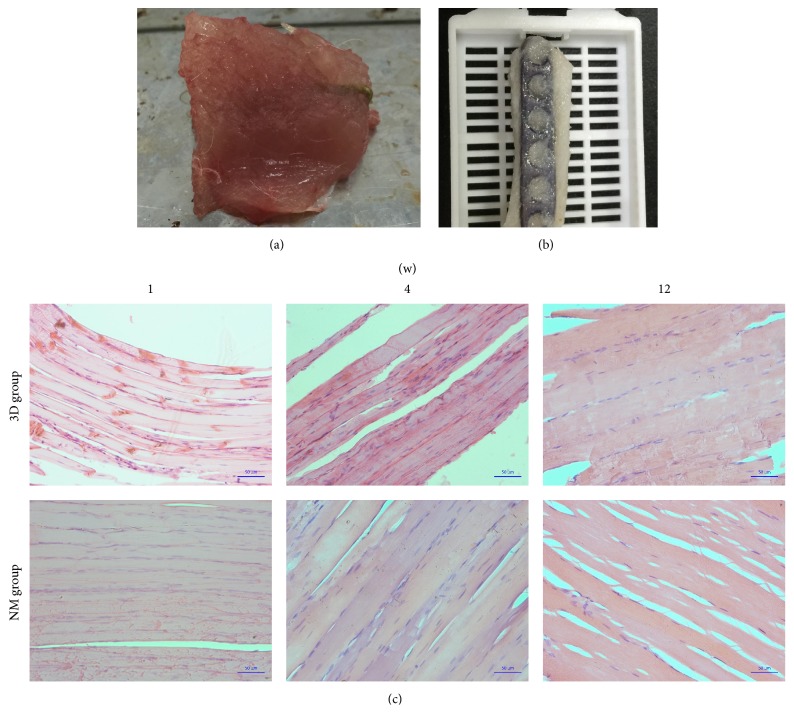
Gross appearance of 3D-printing plate after being implanted for 12 weeks; (a) the plate was surrounded by muscular tissue; (b) the plate and muscular tissue after 4% paraformaldehyde fixed; examination under microscope of muscular tissue by HE staining (200x); (c) the microscopic structure of the muscular tissue of 3DP group and the microscopic structure of the muscular tissue of NM group.

**Figure 9:**
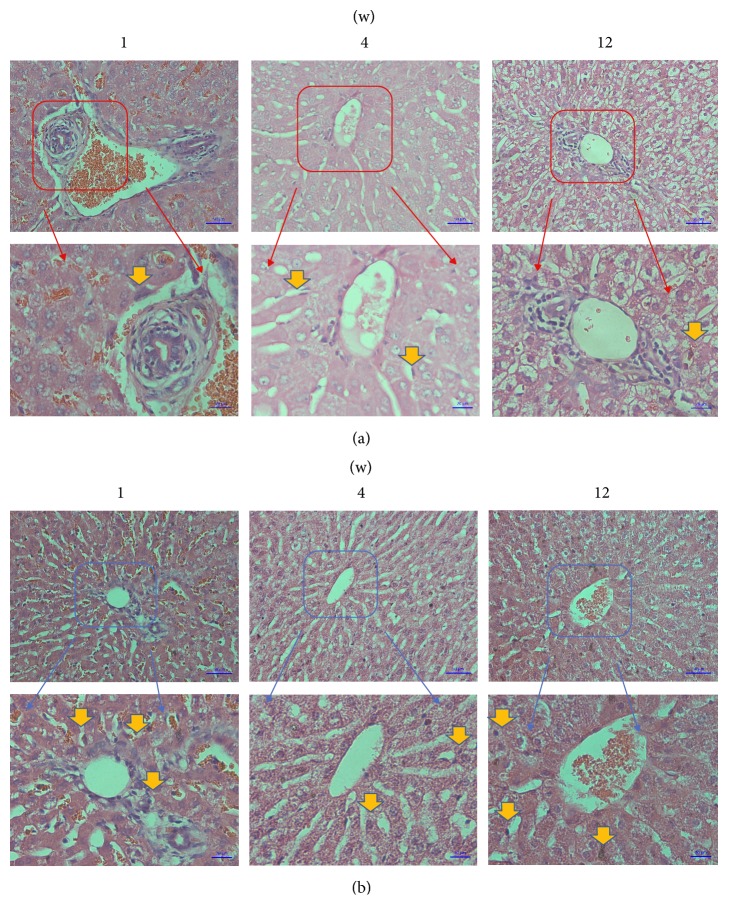
Examination under microscope of liver tissue by HE staining (200x); (a) the microscopic structure of the liver tissue of 3DP group; (b) the microscopic structure of the liver tissue of NM group. Yellow arrows indicate some inflammatory cells.

**Figure 10:**
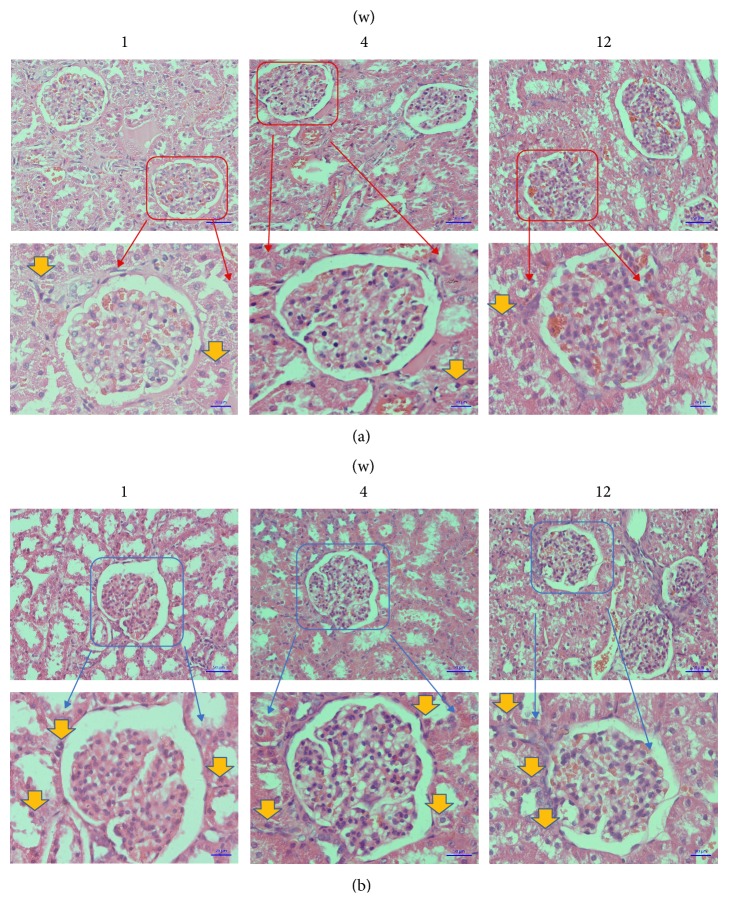
Examination under microscope of kidney tissue by HE staining (200x); (a) the microscopic structure of the kidney tissue of 3DP group; (b) the microscopic structure of the kidney tissue of NM group. Yellow arrows indicate some inflammatory cells.

The general observations and histological measurements were revealed at 1, 4, and 12 weeks after transplantation. The wound healing of each rabbit was great in each group, respectively. Moreover, the observation of the muscles around the plates showed that, in the 3D-printed group, the plates were wrapped by muscle tissue and showed no evidence of cystic cavity or other inflammatory reactions. The muscle fibers in the 3D-printed group were partly broken but still maintained a basic fiber bundle form, and normal arrangement of muscle cells with a homogeneous cytoplasm and clear nucleus, with no inflammatory cell infiltration. There was no significant difference between the NM group and the 3D-printed group (Figure 8). No accumulation of inflammatory cells or structural changes was observed from pathological section of 3D-printed group, similar to the NM group (Figure 9). Similar to the kidney tissue in the NM group, no tissue damage or inflammatory changes were observed from the 3D-printed group (Figure 10).

## 4. Discussion

At present, due to the shape of current acetabular fracture plates that do not meet the clinical need, as they do not fit closely to the fracture, the duration of surgery has to be prolonged for intraoperative bending of the plate, increasing surgical risk. Thus, as one of the most promising directions in 3D-printing technology [14, 15], personalized 3D-printing is considered an effective way of solving the problems described above [16, 17].

Most studies focus on clinical applications of metal 3D-printed implants [18, 19], while few studies explore whether the implants are appropriate for clinical application. Although the titanium alloy powder in the composition is nominally the same as that in a conventional plate that was forged, the change in manufacturing process causes a difference in the properties of the 3D-printed materials, with unknown biocompatibility. Thus we decided to test the biocompatibility of 3D-printed titanium alloy plates both* in vitro* and* in vivo* and compare it to titanium alloy (Ti6AL6V) plates widely used in clinic [20].

Heavy metal ions may be precipitated from titanium alloy plates, as shown by Chaturvedi [21], including titanium and vanadium ions that have significant cytotoxicity to cells, manifesting as rupture of the cell membrane and damage to organelles, leading to cell necrosis. In this study, the cytotoxicity test demonstrated that extracts from 3D-printed titanium alloy plates had no obvious side effects on mouse fibroblasts and no statistically significant difference between 3D-printed and traditional titanium alloy plates. This result is similar to previous studies on cytotoxicity tests for titanium alloy implants [22]. Shah et al. reported that the contact surface between 3D-printed titanium alloy (Ti6AL4V) plates and the bone can promote bone maturation, demonstrating the excellent cellular compatibility of 3D-printed titanium alloy plates [23]. In this study, we observed that cells were correctly formed and were in good condition during coculture of 3D-printed plate extracts and cells (Figure 3). A preliminarily analysis suggests that laser melting deposition causes more tight metal ion binding with a narrower metal ion gap and smaller degree of precipitation of heavy metal ions and so no toxic side effects on cells.

The results of the* in vitro* tests demonstrated good biocompatibility, suggesting that the 3D-printed implants could be further analyzed* in vivo* to assess their host-response. In this study, a sensitization test, systemic toxicity test, and muscle implantation test were performed. The results demonstrated that extracts from 3D-printed plates did not cause allergic reactions to the skin of guinea pigs, which was consistent with the results of the maximum dose sensitization test of a new porous titanium-nickel alloy in guinea pigs reported by Assad et al. [24]. As the first cause of an immune response in allergic reactions is antigen presentation, metal ions precipitated from 3D-printed plates can act as an activator. However, the results of the sensitization test showed that the metal ions released from the surface of 3D-printed plates did not cause immune complex reaction, attributed to the laser melting technology that melts metal powder by high temperature, making the metal ions more closely linked and more difficult to ionize.

In this study, the ionization of plates could be simulated* in vivo* through extracts from 3D-printed plates, according to the relevant ISO standard [13]. The extracts were injected into C57 mice through their tail veins, and acute toxicity was measured over a short period of time by observing the activity and weight change in the mice over 3 days, so as to assess the safety of the plate, as shown previously by Zamora et al. [25]. The results of this study are consistent with those of He et al. [26] in that 3D-printed bone scaffold extracts in the systemic circulation appeared to induce no obvious toxicity.

When implants are placed within a host, the immune system responds, and so it can be determined whether a foreign body will induce toxic effects on the body. Once considered to be destructive molecules, inflammatory mediators will be induced to form by the immune system around the implant, inhibiting further negative impact on the body [27, 28]. In previous studies, Shah et al. [29] pointed out that titanium alloy plates manufactured by laser melting technology were implanted in experimental animals, which had not had their immune response induced significantly. The results of this test were in broad agreement with the results of the muscle implant test in this study. The metal ions are ionized or dissolved from the implants in the body and pass into the blood and then they are detoxified in the liver and finally eliminated through the urinary system. In this process, the liver and the kidney are more vulnerable to the toxic effects of metal ions. Thus, in order to observe the effect of precipitation of metal ions on the body, pathological examination of the animals' livers and kidneys is critical to determining implant safety. Li et al. [30] constructed porous titanium alloy stents by 3D-printing technology and implanted them into goats, which showed that pathological analyses of the livers and kidneys in the experimental animals were consistent with those of normal animals during the longest 12-week period, with no obvious signs of damage or necrosis. The results obtained in this study were similar, with 3D-printed plates fabricated using laser melting technology. The technology involves the metal powder rapidly melting and solidifying. As the powder contains Al, V, O, H, N, C, and other alloy elements, small intermetallic compounds can be formed after laser heating, so that metal ions are firmly locked into the implant without being precipitated.

We performed both* in vitro* and* in vivo* tests, which simulated implantation into the internal environment. Based on those results, we evaluated the biocompatibility of 3D-printed titanium plates. The biocompatibility was evaluated using many techniques from extracts formed* in vitro* and* in vivo* by intradermal and intravenous injection and by* in vivo* intramuscular implantation, which was able to establish whether the implants were biocompatible with comprehensive information and convincing evidence. The downside was that the methods used are generic and failed to provide an innovative approach.

## 5. Conclusions

Metal 3D-printing is a highly accurate processing and speed-manufacturing technique which makes the manufacture of single articles with personalized design and complicated structures suitable. The aim of our research was to apply this emerging technology to fabricate personalized-designed plates for the treatment of acetabular fracture. After manufacturing plates by 3D-printing in metal, we performed further studies. In this part, our laboratory findings showed favorable biocompatibility of 3D-printed titanium alloy plates. It may provide some novelty for providing solutions for the clinical treatment of acetabular fracture.

## Figures and Tables

**Table 1:** Cell OD values in each group at different times (hours).

Groups	24 h	72 h	120 h
100% 3D	0.3483 ± 0.0456	0.4397 ± 0.0190	0.6050 ± 0.0180
50% 3D	0.3602 ± 0.0165	0.4867 ± 0.0360	0.5957 ± 0.0200
25% 3D	0.3926 ± 0.0178	0.4793 ± 0.0270	0.6163 ± 0.0190
0% 3D	0.3700 ± 0.0358	0.4567 ± 0.0220	0.6180 ± 0.0430
NM	0.3840 ± 0.0614	0.4577 ± 0.0350	0.6117 ± 0.0120

**Table 2:** RGR (relative growth rate) in each group at different times (hours).

Groups	24 h	72 h	120 h
100% 3D	0.7265	0.9128	0.9799
50% 3D	0.8171	1.1405	0.9518
25% 3D	1.0658	1.1050	1.0141
0% 3D	0.8923	0.9952	1.0191
NM	1.0000	1.0000	1.0000
